# Report from the American Society for Microbiology COVID-19 International Summit, 23 March 2020: Value of Diagnostic Testing for SARS–CoV-2/COVID-19

**DOI:** 10.1128/mBio.00722-20

**Published:** 2020-03-26

**Authors:** Robin Patel, Esther Babady, Elitza S. Theel, Gregory A. Storch, Benjamin A. Pinsky, Kirsten St. George, Tara C. Smith, Stefano Bertuzzi

**Affiliations:** a Mayo Clinic, Rochester, Minnesota, USA; b Memorial Sloan Kettering Cancer Center, New York, New York, USA; c Washington University, Saint Louis, Missouri, USA; d Stanford University School of Medicine, Palo Alto, California, USA; e Wadsworth Center, New York State Department of Health, Albany, New York, USA; f Kent State University, Kent, Ohio, USA; g American Society for Microbiology, Washington, DC, USA

## GUEST EDITORIAL

As we enter the second quarter of the COVID-19 pandemic, with testing for severe acute respiratory syndrome coronavirus 2 (SARS–CoV-2) increasingly available (though still limited and/or slow in some areas), we are faced with new questions and challenges regarding this novel virus. When to test? Whom to test? What to test? How often to test? And, what to do with test results? Since SARS–CoV-2 is a new virus, there is little evidence to fall back on for test utilization and diagnostic stewardship (1). Several points need to be considered to begin answering of these questions; specifically, what types of tests are available and under which circumstances are they useful? This understanding can help guide the use of testing at the local, regional, state, and national levels and inform those assessing the supply chain to ensure that needed testing is and continues to be available. Here, we explain the types of tests available and how they might be useful in the face of a rapidly changing and never-before-experienced situation. There are two broad categories of SARS–CoV-2 tests: those that detect the virus itself and those that detect the host’s response to the virus. Each will be considered separately.

We must recognize that we are dealing with (i) a new virus, (ii) an unprecedented pandemic in modern times, and (iii) uncharted territory. With this in mind, in the absence of either proven effective therapy or a vaccine, diagnostic testing, which we have, becomes an especially important tool, informing patient management and potentially helping to save lives by limiting the spread of SARS–CoV-2. What is the most appropriate test, and for whom and when?

Hypothetically, if the entire world’s population could be tested all at once, with a test providing 100% specificity and sensitivity (unrealistic, obviously), we might be able to identify all infected individuals and sort people into those who at that moment in time were asymptomatic, minimally/moderately symptomatic, and severely symptomatic. The asymptomatic and minimally/moderately symptomatic could be quarantined to avoid the spread of the virus, with the severely symptomatic managed and isolated in health care settings. Contract tracing could be carried out to find those at risk of being in the incubation period by virtue of their exposure. Alternatively, testing for a host response, if, again, the test were hypothetically 100% sensitive and specific, could identify those previously exposed to the virus and (if we knew this to be true, which we do not) label those who are immune to the virus, who could be tapped to work in settings where potentially infected individuals (e.g., sick patients in hospitals) might otherwise pose a risk. Unfortunately, these hypothetical scenarios are not reality. However, with this ideal situation as a guide, what we do have available as tests today should be carefully considered in terms of how they can be leveraged to move the current crisis closer to the ideal situation, especially in the absence of therapeutics or vaccines.

Although the virus can be cultured, this is dangerous and not routinely done in clinical laboratories. While detection of viral antigens is theoretically possible, this approach has not, to date, been a primary one, but one that those participating in the summit considered to deserve further research.

## TEST 1. TESTS FOR VIRAL RNA

Most tests currently used for direct detection of SARS–CoV-2 identify viral RNA through nucleic acid amplification, usually using PCR. An important consideration is exactly what gets tested for viral RNA. Tests that detect viral RNA are contingent on viral RNA being present in the sample collected. The most common sample types being tested are swabs taken from the nasopharynx and/or oropharynx, with the former considered somewhat more sensitive than the latter (2); if both are collected, the two swabs may be combined and tested simultaneously in a single reaction to conserve reagents. Today, health care professionals collect these swabs; however, evidence suggests that patients or parents (in the case of young children) might be able to collect their own swabs (3, 4). Following collection, swabs are placed into a liquid to release virus/viral RNA from the swabs into solution. Then, viral RNA is extracted from that solution and subsequently amplified (e.g., by reverse transcription-PCR).

**FIG 1:**
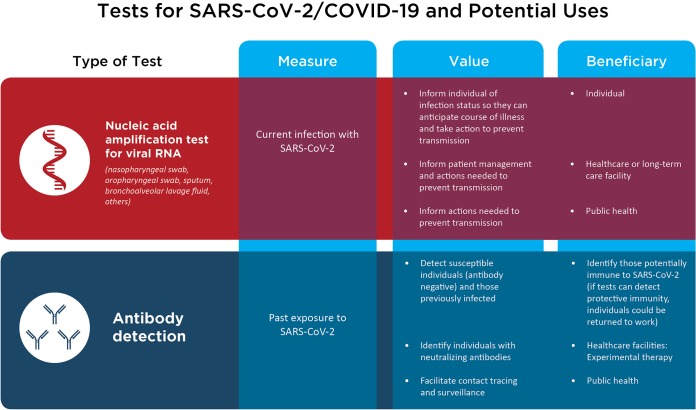
Tests for SARS–CoV-2/COVID-19 and potential uses.

For patients with pneumonia, in addition to nasopharyngeal and oral secretions, lower respiratory tract secretions, such as sputum and bronchoalveolar lavage fluid, are tested. It should not be assumed that each of these (e.g., nasopharyngeal swab specimen, sputum, bronchoalveolar lavage fluid) will have the same chance of detecting SARS–CoV-2; detection rates in each sample type vary from patient to patient and may change over the course of individual patients’ illnesses. Some patients with pneumonia may have negative nasal or oropharyngeal samples but positive lower airway samples (5), for example. Accordingly, the true clinical sensitivity of any of these tests is unknown (and is certainly not 100%, as in the hypothetical scenario); a negative test does not therefore negate the possibility that an individual is infected. If the test is positive though, the result is most likely correct, although stray viral RNA that makes its way into the testing process (for example, as the specimen is being collected or as a result of specimen cross-contamination or testing performed by a laboratory worker who is infected with SARS–CoV-2 [these are just some examples]) could conceivably result in a falsely positive result. Also, we note that viral RNA does not equate to live virus, and therefore, detection of viral RNA does not necessarily mean that the virus can be transmitted from that patient. That said, viral RNA-based tests are the best tests that we have in the setting of an acute illness. It is important to recognize that the accuracy of the test is affected by the quality of the sample, and thus it is critical that the sample be obtained in a proper (and safe) manner. Testing patients for SARS–CoV-2 helps identify those who are infected, which is useful for individual patient management, as well as for implementation of mitigation strategies to prevent spread in health care facilities and in the community alike (Fig. 1).

There are numerous unanswered questions, challenges, and controversies surrounding testing for viral RNA. RNA may degrade over time. There are concerns that specimen collection for testing is exhausting the supply of critical personal protective equipment needed to care for infected patients. Alternative strategies for specimen collection, including home collection, should therefore be considered either by a health care provider or patients themselves (or a parent in the case of young children); the use of alternative specimen types, such as oral fluid or nasal swabs (if they are shown to provide results equivalent to those from nasopharyngeal swabs) should also be considered. Spread to health care workers and within health care and long-term-care facilities is a primary consideration for prioritization of testing; testing of patients likely to have SARS–CoV-2 who are in health care facilities or long-term-care facilities, alongside potentially ill workers critical to the pandemic response, including health care workers, public health officials, and other essential leaders, is a priority. That said, testing anyone who has symptoms compatible with COVID-19 should be considered, since broad testing will help define who has this infection, allowing control of its spread. Given that SARS–CoV-2 can infect anyone and result in transmission prior to the onset of symptoms or even possibly without individuals ever developing symptoms, testing asymptomatic patients could even be considered. Unfortunately, little is known at this time about viral RNA detection in asymptomatic patients, and such testing strategies may stretch available resources beyond realistic limits. Some future therapeutics may work best if given early, which will demand early testing for SARS–CoV-2 to realize maximal efficacy. The questions of how many tests are needed and what kind should be performed on individual patients (for primary diagnosis if results of initial testing are negative and subsequently to document clearance of the virus to release patients from isolation) remain open.

As the number of tests available for SARS–CoV-2 increases, new challenges, including the needs to (i) better understand variability in the performance characteristics of the various tests (e.g., sensitivity and specificity), including on different samples types, (ii) optimize assays from their original design (e.g., multiple targets to a single target) to improve reagent utilization while maintaining performance characteristics, and (iii) monitor test performance given the potential for the virus to mutate, are emerging. The last can be addressed by periodically sequencing the evolved virus to look for changes in primer and probe binding regions that might affect the performance of tests based on the detection of viral RNA; periodic sequencing can also aid in tracking viral evolution. Additionally, as testing increases, decreasing the time to results of testing will continue to be crucial to better manage both patients and health care workers. Development of rapid, point-of-care diagnostics is a gap and should be a priority. Measurement of viral levels may also be useful to monitor recovery, response to therapy, and/or level of infectivity. Current RNA-based diagnostic tests are primarily qualitative, and although they could be calibrated to provide viral loads, a standardized process does not currently exist. Of note, there is no established threshold for interpretation of viral loads, which may vary in different hosts.

Although tests have become available, the huge demand for them has created supply chain challenges, compromising their very availability; this includes issues with the availability of nasopharyngeal swabs, RNA extraction reagents and instruments, and PCR reagents and instruments. Even with now-FDA-approved/cleared commercial tests, there are delays with the installation of instruments and supply of reagents/kits to meet the demand at many sites. At the moment, extensive efforts are being made on multiple fronts to address the numerous supply challenges surrounding testing and a secure continuity of testing services.

## TEST 2. SEROLOGY

The other broad category of tests is those that detect IgM, IgA, IgG, or total antibodies (typically in blood). Development of an antibody response to infection can be host dependent and take time; in the case of SARS–CoV-2, early studies suggest that the majority of patients seroconvert between 7 and 11 days postexposure to the virus, although some patients may develop antibodies sooner. As a result of this natural delay, antibody testing is not useful in the setting of an acute illness. We do not know for certain whether individuals infected with SARS–CoV-2 who subsequently recover will be protected, either fully or partially, from future infection with SARS–CoV-2 or how long protective immunity may last; recent evidence from a rhesus macaque study does suggest protective immunity after resolution of a primary infection (https://doi.org/10.1101/2020.03.13.990226); however, further studies are needed to confirm this. Antibody tests for SARS–CoV-2 may facilitate (i) contact tracing—RNA-based tests can help with this as well; (ii) serologic surveillance at the local, regional, state, and national levels; and (iii) identification of those who have already had the virus and thus may (if there is protective immunity) be immune. Assuming there is protective immunity, serologic information may be used to guide return-to-work decisions, including for individuals who work in environments where they can potentially be reexposed to SARS–CoV-2 (e.g., healthcare workers). Serologic testing may also be useful to identify individuals who may be a source for (currently experimental) therapeutic or prophylactic neutralizing antibodies. In addition, antibody testing can be used in research studies to determine the sensitivity of PCR assays for detecting infection and be employed retrospectively to determine the true scope of the pandemic and assist in the calculation of statistics, including the case fatality rate. Finally, serologic testing can possibly be used diagnostically to test viral RNA-negative individuals presenting late in their illness.

Summit participants noted that testing for host markers might be needed to fully understand which patients are at risk of developing severe disease from their infection.

In summary, both of the two categories of tests for SARS–CoV-2 should be useful in this outbreak. We are fortunate to have the technologies we do that have allowed diagnostics to be made rapidly available. There is likely to be a direct connection between understanding the level of virus/disease in individual communities and acceptance of control measures that require individual action, such as social distancing. Now, we need to ensure systematic and coordinated efforts between the public, clinical, commercial, and industry sectors to ensure robust supply lines in the midst of the pandemic so that we can leverage the power of testing to address the pandemic confronting us.
